# Structural characterization of angiotensin I-converting enzyme in complex with a selenium analogue of captopril

**DOI:** 10.1111/j.1742-4658.2011.08276.x

**Published:** 2011-10

**Authors:** Mohd Akif, Geoffrey Masuyer, Sylva L U Schwager, Bhaskar J Bhuyan, Govindasamy Mugesh, R Elwyn Isaac, Edward D Sturrock, K Ravi Acharya

**Affiliations:** 1 Department of Biology and Biochemistry, University of BathUK; 2 Division of Medical Biochemistry and Institute of Infectious Disease and Molecular Medicine, University of Cape TownObservatory, South Africa; 3 Department of Inorganic and Physical Chemistry, Indian Institute of ScienceBangalore, India; 4 Institute of Integrative and Comparative Biology, University of LeedsUK

**Keywords:** angiotensin I-converting enzyme (ACE), cardiovascular disease, inhibitor design, metalloprotease, selenium

## Abstract

Human somatic angiotensin I-converting enzyme (ACE), a zinc-dependent dipeptidyl carboxypeptidase, is central to the regulation of the renin–angiotensin aldosterone system. It is a well-known target for combating hypertension and related cardiovascular diseases. In a recent study by Bhuyan and Mugesh [*Org. Biomol. Chem.* (2011) **9**, 1356–1365], it was shown that the selenium analogues of captopril (a well-known clinical inhibitor of ACE) not only inhibit ACE, but also protect against peroxynitrite-mediated nitration of peptides and proteins. Here, we report the crystal structures of human testis ACE (tACE) and a homologue of ACE, known as AnCE, from *Drosophila melanogaster* in complex with the most promising selenium analogue of captopril (SeCap) determined at 2.4 and 2.35 Å resolution, respectively. The inhibitor binds at the active site of tACE and AnCE in an analogous fashion to that observed for captopril and provide the first examples of a protein–selenolate interaction. These new structures of tACE–SeCap and AnCE–SeCap inhibitor complexes presented here provide important information for further exploration of zinc coordinating selenium-based ACE inhibitor pharmacophores with significant antioxidant activity.

**Database:**

Structural data for the two SeCap complexes with ACE and AnCE have been deposited with the RCSB Protein Data Bank under the codes 2YDM and 3ZQZ, respectively.

## Introduction

Human angiotensin I-converting enzyme (ACE; EC3.4.15.1) is a zinc metallopeptidase that plays a critical role in blood pressure regulation [1–7] by catalysing the proteolysis of angiotensin I to the vasopressor angiotensin II [8–10]. There are two isoforms of human ACE: in somatic tissues, it exists as a glycoprotein composed of a mature single polypeptide chain of 1277 amino acids with two active centres, one in each of the N- and C-domains [11]. Testis ACE (tACE) is identical to the C-terminal half of somatic ACE, except for a unique 36-residue sequence at its N-terminus [12]. Both domains are heavily glycosylated (the N-domain has 10 and the C-domain has 7 N-linked glycosylation sites), cleave angiotensin I, are dependent on chloride ion activation and share ∼ 55% amino acid sequence identity.

ACE inhibitors are widely used in clinical practice for the treatment of hypertension, heart failure, myocardial infarction and diabetic nephropathy. In addition, a number of studies have suggested that hypertension and oxidative stress are interdependent [13,14]. Therefore, ACE inhibitors having antioxidant properties are considered beneficial for the treatment of hypertension. Because selenium compounds are known to exhibit better antioxidant behaviour than their sulfur analogues [15,16], we have recently reported the synthesis, characterization and antioxidant activity of a number of selenium analogues of the clinically used ACE inhibitor captopril [17,18]. It was shown that selenium analogues of captopril (SeCap) not only inhibit ACE activity, but also can effectively scavenge peroxynitrite, a strong oxidant found *in vivo* [19].

The two ACE homologues, AnCE and ACER, from a nonvertebrate, *Drosophila melanogaster*, have been studied in detail. AnCE is a single-domain protein, reported to have biochemical resemblance to C-domain ACE [20]. In addition the 3D structure(s) of native AnCE and AnCE in complex with ACE inhibitors has firmly established the high degree of conservation in the active site of ACE [21,22].

**Fig. 1:**
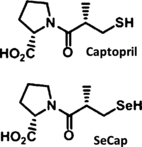
Chemical structures of captopril and SeCap.

Here, we report for the first time, structural details on the binding of one of the potent SeCaps (Fig. 1) to tACE and AnCE, as elucidated by X-ray crystallography at 2.4 and 2.35 Å resolution, respectively. Using these structures, we have been able to make a direct comparison of the previously determined structures of native tACE [23] and AnCE [22] with their respective complex with captopril [22,24]. These structures are useful in understanding a selenolate ligand's coordination of zinc and its binding mode at the active site of ACE and its homologue AnCE.

## Results and Discussion

### tACE–SeCap complex

**Fig. 2:**
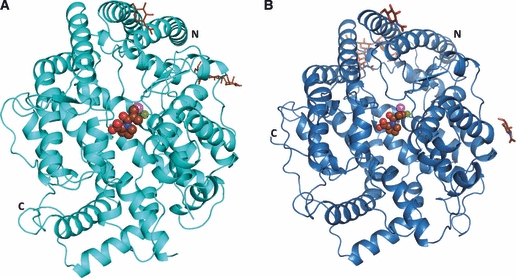
(A) Structure of tACE (cyan) with inhibitor SeCap bound at the active site cavity (shown in spheres). The zinc ion (green sphere) bound in the active site and *N*-glycosylated sugars (brown sticks) at potential sites, Asn72, Asn109 are shown. Protein termini are labelled. (B) Structure of AnCE (blue) with inhibitor SeCap bound at the active site cavity (shown in spheres). The zinc ion (green sphere) bound in the active site and *N*-glycosylated sugars (brown sticks) at potential sites, Asn53, Asn196, Asn311 are shown.

**Fig. 3:**
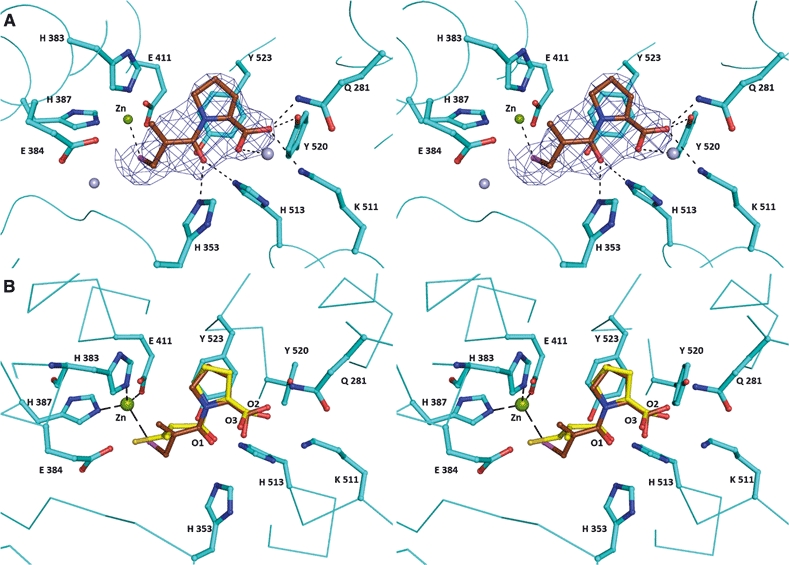
(A) A stereo representation of tACE active site with bound inhibitor. The inhibitor molecule is shown in a stick model (brown) with the electron density map contoured at 1σ level. The zinc ion is shown as a green sphere and water molecules in light blue colour. Interacting residues are labeled and atoms are coloured as follows: red for oxygen, blue for nitrogen and purple for selenium. Hydrogen bonds are shown as dotted lines. (B) Comparison of SeCap (this study, left) and captopril (yellow sticks) binding [24] to tACE (right).

The final structure contains a zinc ion, one SeCap inhibitor molecule, N-glycosylated carbohydrates at two potential binding sites (Asn72, Asn109) and 54 water molecules (Table 1). No noticeable conformational change in the protein is observed upon inhibitor binding. The topological arrangement of the final structure (Fig. 2A) is consistent with previously determined structures of tACE [23], as well as tACE in complex with captopril [24]. One bound SeCap molecule was unambiguously fitted in the catalytic site of tACE (Fig. 3A) with the aid of a clearly observed electron-density map.

**Table 1:** X-ray diffraction data collection and refinement statistics. Values in parentheses are for last resolution shell. SeCap, selenium analogue of captopril; tACE, testis angiotensin I converting enzyme

	tACE–SeCap inhibitor complex	AnCE–SeCap inhibitor complex
Resolution (Å)	2.4	2.35
Space group	*P2*_*1*_*2*_*1*_*2*_*1*_ (one molecule/asymmetric unit)	*R3* (one molecule/asymmetric unit)
Cell dimension (Å, deg)	*a* = 56.1, *b* = 84.5, *c* = 132.3 α = β = γ = 90	*a* = 173.8, *b* = 173.8, *c* = 100.8 α = β = 90, γ = 120
Total no. of observations	117329	179815
No. of unique reflections	23627	44663
Completeness (%)	97.4 (83.6)	94.7 (72.1)
*I/σ(I)*	12.3 (2.2)	11.5 (1.6)
*R*_symm_^a^	0.09 (0.62)	0.075 (0.53)
*R*_cryst_^b^*/R*_free_^c^	0.21/0.26	0.20/0.24
No. of protein atoms	4687	4866
No. of solvent atoms	54	201
No. of inhibitor atoms	14	14
Deviation from ideality		
Bond lengths (Å)	0.01	0.01
Bond angles (deg)	1.42	0.88
B-factor analysis		
Protein all atoms	32.6	35.3
Protein main chain	32.2	35.2
Protein side chain	33.0	35.3
Solvent atoms	32.3	35.4
Inhibitor atoms	46.9	51.0
Zn^2+^/Cl^−^ ions (tACE); Zn^2+^ ion (AnCE)	35.7/44.0	33.9
Glycosylated sugars	52.7	54.0

a *R*_symm_ = Σ_*h*_Σ_*i*_[|*I*_*i*_(*h*) − <I(*h*)>|/Σ_*h*_Σ_*i*_*I*_*i*_(*h*)], where *I*_*i*_ is the *i*th measurement and *<I*(*h*)*>* is the weighted mean of all the measurements of *I(h)*.

b *R*_cryst_ = Σ_*h*_*|F*_*o*_ − *F*_*c*_*|/*Σ_*h*_*F*_*o*_, where *F*_o_ and *F*_c_ are observed and calculated structure factor amplitudes of reflection *h*, respectively.

c *R*_free_ is equal to *R*_cryst_ for a randomly selected 5% subset of reflections.

The inhibitor SeCap molecule makes a direct interaction with the catalytic Zn^2+^ ion (distance 2.5 Å, Table 2) deep inside the active site channel (Fig. 2A), similar to the zinc coordination in tACE–captopril [24]. This interaction results in the formation of a zinc–selenolate complex. The inhibitor is anchored through the central carbonyl group and the proline carboxylate group. The proline residue of SeCap interacts with the S_2_′ subsite of the active site via two strong hydrogen bonds from two histidines (His513, 3.1 Å; His353, 2.6 Å). One oxygen atom of the proline carboxylate group is held by interactions with Tyr520 (2.7 Å), Gln281 (2.7 Å) and Lys511 (3.0 Å) (Fig. 3A, Table 2). In addition, it is held by seven hydrogen bonds including two mediated through water molecules, as calculated by hbplus [25] (Table 2). Thus, the interactions of SeCap with tACE residues are almost identical to those observed with captopril [24] (Fig. 3B), which is not too surprising considering the similarity between the two chemical structures (Fig. 1).

**Table 2:** Hydrogen bond contacts of testis angiotensin I converting enzyme (tACE) with the selenium analogue of captopril (SeCap) inhibitor

	Atom	Inhibitor atom	Distance (Å)
tACE Residue			
His353	NE2	O1	2.6
His513	NE2	O1	3.1
Gln281	NE2	O2	2.7
Lys511	NZ	O2	3.0
Tyr520	OH	O2	2.7
Zinc ion			
	Zn	Se	2.5
Water molecule			
	O	O3	3.0
	O	Se	3.4

### AnCE–SeCap complex

**Fig. 4:**
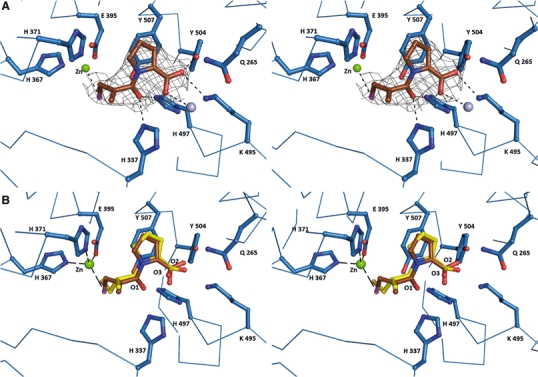
(A) A stereo representation of AnCE active site with bound inhibitor. The inhibitor molecule is shown in a stick model (brown) with the electron density map contoured at 1σ level. The zinc ion is shown as a green sphere and water molecules in light blue colour. Interacting residues are labeled and atoms are coloured as follows: red for oxygen, blue for nitrogen and purple for selenium. Hydrogen bonds are shown as dotted lines. (B) Comparison of SeCap (this study, left) and captopril (yellow sticks) binding [22] to AnCE (right).

The final structure contains a zinc ion, one inhibitor molecule, N-glycosylated carbohydrates at three potential binding sites (Asn53, Asn196, Asn311) and 201 water molecules (Table 1). No noticeable conformational change is observed upon inhibitor binding. The topological arrangement of the final structure (Fig. 2B) is consistent with previously determined structures of AnCE and AnCE in complex with captopril [21,22]. One bound SeCap molecule was unambiguously fitted in the catalytic site of AnCE (Fig. 4A) with the aid of a clearly observed electron-density map.

The inhibitor SeCap molecule makes a direct interaction with the catalytic Zn^2+^ ion, displacing the water that is bound in the native enzyme (distance 2.7 Å, Table 3) (Fig. 4A), similar to the zinc coordination in AnCE–captopril [21,22]. This interaction results in the formation of a zinc–selenolate complex. The carboxy-end of the proline moiety and the central carbonyl interact with the extended S_1_′S_2_′-binding site locking the inhibitor in position. The proline residue of SeCap makes important contacts with the S2′ subsite via two strong hydrogen bonds from two histidines (His497, 3.1 Å; His337, 2.5 Å). One oxygen atom of the proline carboxylate group interacts with Tyr504 (2.5 Å), Gln265 (2.9 Å) and Lys495 (2.9 Å) through hydrogen and ionic bonds (Fig. 4A, Table 3). The central carbonyl of SeCap is held by His337 and His497. In addition, it is held by seven hydrogen bonds, including two hydrogen bonds mediated through water molecules, as calculated by hbplus [25] (Table 3). Thus, the interactions of SeCap with AnCE residues are almost identical to those observed with captopril [21,22] (Fig. 4B).

**Table 3:** Hydrogen bond contacts of AnCE with the selenium analogue of captopril (SeCap) inhibitor

	Atom	Inhibitor atom	Distance (Å)
AnCE residue			
Gln265	NE2	O2	2.9
His337	NE2	O1	2.5
Lys495	NZ	O2	2.9
His497	NE2	O1	3.1
Tyr504	OH	O2	2.5
Zinc ion			
	Zn	Se	2.7
Water molecule			
	O	O3	2.9
	O	O3	3.5

## Conclusions

There has been considerable interest in applications of selenolate compounds because of their broad therapeutic spectrum and low toxicity. The organoselenium compound ebselen [2-phenyl-1,2-benzisoselenazol-3(2H)-one] an anti-inflammatory and general antioxidant, is also a potent inhibitor of extracellular nucleoside diphosphokinase [26]. Furthermore, Achillion Pharmaceuticals (New Haven, CT, USA) has recently developed a selenophene inhibitor of bacterial topoisomerases that shows promise as an antibiotic [27]. However, selenolates have not been exploited as metalloprotease inhibitors despite their antioxidant- and peroxynitrite-scavenging activities. This study has, for the first time, provided molecular details on the binding and coordination of a selenium analogue of the potent ACE inhibitor captopril with ACE and its homologue AnCE. Captopril is one of the smallest ACE inhibitors and with both ACE and AnCE, key interactions of the selenium analogue's central carbonyl and proline carboxylate anchor the inhibitor in the cavernous S_1_′S_2_′-binding site of the enzyme. The envelope of space surrounding the P_2_′ proline of SeCap is further illustrated by the accommodation of silylated captopril analogues in the active site of ACE [28]. Selenolates are stronger zinc-binding groups than thiols, yet surprisingly the IC_50_ value of SeCap was greater than that of captopril [19]. This anomaly could be due to the cooperativity of the two-domain somatic ACE and the modest N-domain selectivity of captopril. Structures of SeCap complex with the N-domain and somatic ACE will undoubtedly shed further light on the binding of this new zinc-binding group of compounds with ACE and aid in the design of further selenium-based ACE inhibitors.

## Experimental procedures

### tACE–SeCap complex

A variant of tACE (tACEΔ36-g13, underglycosylated protein) was purified to homogeneity from Chinese hamster ovary cells [29]. The inhibitor SeCap (Fig. 1, IC_50_ value of 36.4 ± 1.5 nm) was synthesized as reported recently [19]. A stock solution (5 mm) of SeCap was prepared by dissolving the inhibitor in deoxygenated water containing 10 mm dithiothreitol. The crystals of the tACE complex with SeCap were grown at 16 °C using the hanging drop vapour diffusion method. tACE protein (11.5 mg·mL^−1^ in 50 mm Hepes, pH 7.5) was preincubated with SeCap (1 mm) on ice for 3 h before crystallization. Preincubated sample (2 μL) was mixed with the reservoir solution consisting of 13.5% poly(ethylene glycol) 4000, 50 mm sodium acetate, pH 4.7 and 10 μm ZnSO_4_, and suspended above the well. Diffraction quality of cocrystals appeared after ∼ 10 days.

X-ray diffraction data for the tACE–SeCap complex were collected on the PX station IO2 at Diamond Light Source (Didcot, UK). A total of 150 images were collected using a Quantum-4 CCD detector (ADSC Systems, Poway, CA, USA). No cryoprotectant was used to keep the crystal at constant temperature (100 K) under the liquid nitrogen jet during data collection. Raw data images were indexed and scaled with xds [30] and the CCP4 program scala [31]. Initial phasing for structure solution was obtained using the molecular replacement routines of the program phaser [32]. The atomic coordinates of native tACE [23] (PDB code 1O8A) were used as a search model. The resultant model was refined using refmac5 [33] and adjustment of the model was carried out using coot [34]. Water molecules were added at positions where *F*_o_ − *F*_c_ electron-density peaks exceeded 3σ and potential H-bonds could be made. Based on electron-density interpretation, the inhibitor and sugar moieties were added in the complex structure and further refinement was carried out. The coordinate and parameter files for SeCap were generated using sketcher [31]. Validation was conducted with the aid of molprobity [35]. Figures were drawn with pymol (DeLano Scientific, San Carlos, CA, USA). Hydrogen bonds were verified with the program hbplus [25]. The detailed refinement statistics for the complex structure are given in Table 1.

### AnCE–SeCap complex

AnCE was cloned and expressed in *Pichia pastoris* as described previously [22]. In brief, AnCE was purified to homogeneity from culture media using hydrophobic interaction chromatography and size-exclusion chromatography. The crystals of the AnCE complex with SeCap were grown at 21 °C by the hanging drop vapour diffusion method. AnCE protein (10 mg·mL^−1^ in 50 mm Hepes, pH 7.5) was preincubated with SeCap (1 mm) and 10 μm zinc acetate, on ice for 3 h before crystallization. Preincubated sample (2 μL) was mixed with the reservoir solution consisting of 1.3 m sodium citrate, 100 mm Hepes, pH 7.5 and suspended above the well. Diffraction quality of cocrystals appeared after ∼ 1 week. X-ray diffraction data for the AnCE–SeCap complex were collected on the PX station I24 at Diamond Light Source. A total of 170 images were collected using a PILATUS 6M detector (Dectris, Baden, Switzerland). No cryoprotectant was used. Raw data images were indexed and scaled with xds [30] and the CCP4 program scala [31]. Initial phasing for structure solution was obtained using the molecular replacement routines of the program phaser [32]. The atomic coordinates of native AnCE [22] (PDB code 2X8Y) were used as a search model. The resultant model was refined using refmac5 [33] and adjustment of the model was carried out using coot [34]. Water molecules were added at positions where *F*_*o*_ *− F*_*c*_ electron-density peaks exceeded 3σ and potential H-bonds could be made. Based on electron-density interpretation, the inhibitor and sugar moieties were added in the complex structure and further refinement was carried out. The coordinate and parameter files for SeCap were generated using sketcher [31]. Validation was conducted with the aid of molprobity [35]. Figures were drawn with pymol. Hydrogen bonds were verified with the program hbplus [25]. The detailed refinement statistics for the complex structure are given in Table 1.

## Abbreviations

ACE: angiotensin I-converting enzyme
SeCap: selenium analogue of captopril
tACE: testis angiotensin I-converting enzyme
